# Supplementary material for: Nationwide increases in anti-SARS-CoV-2 IgG antibodies between October 2020 and March 2021 in the unvaccinated Czech population
Source: Commun Med (Lond). 2022 Mar 1;2:19. doi: 10.1038/s43856-022-00080-0 (PMC9053194; doi:10.1038/s43856-022-00080-0)
Supplement: Supplementary file 1 — Description of Additional Supplementary Files [file 43856_2022_80_MOESM1_ESM.pdf]

Description of Additional Supplementary Files

File Name: Supplementary Data 1

Description: Prevalence rate ratios (PRRs) and 95 % confidence intervals for seroprevalence of IgG antibodies to SARS-CoV-2 by BMI categories and test provider in PROSECO study participants estimated by multivariate Poisson regression

File Name: Supplementary Data 2

Description: Source data underlying Figure 1: Dynamics of the COVID-19 pandemic in the Czech Republic and seroprevalence in the first phase of the PROSECO study between October 2020 and March 2021
