# Supplementary material for: Nationwide increases in anti-SARS-CoV-2 IgG antibodies between October 2020 and March 2021 in the unvaccinated Czech population
Source: Commun Med (Lond). 2022 Mar 1;2:19. doi: 10.1038/s43856-022-00080-0 (PMC9053194; doi:10.1038/s43856-022-00080-0)
Supplement: Supplementary file 2 — Supplementary Data 1 [file 43856_2022_80_MOESM2_ESM.docx]

**Supplementary table 1:** Prevalence rate ratios (PRRs) and 95 % confidence intervals for seroprevalence of IgG antibodies to SARS-CoV-2 by BMI categories and test provider in PROSECO study participants estimated by multivariate Poisson regression

|  | **October 2020 – November 2020 n = 3,626** | | | | **December 2020 – January 2021**  **n = 6,880** | | | | **February 2021 – March 2021**  **n = 19,548** | | | | **All study periods**  **n = 30,054** | | | |
| --- | --- | --- | --- | --- | --- | --- | --- | --- | --- | --- | --- | --- | --- | --- | --- | --- |
|  | **Particip.^1^ n**  **(%)** | **Seropositive^2^ (%)** | **PRR**  **(95% CI)** | **p-value** | **Particip.^1^**  **n**  **(%)** | **Seropositive^2^**  **(%)** | **PRR**  **(95% CI)** | **p-value** | **Particip. ^1^**  **n**  **(%)** | **Seropositive^2^**  **(%)** | **PRR**  **(95% CI)** | **p-value** | **Particip. ^1^**  **n**  **(%)** | **Seropositive^2^**  **(%)** | **PRR**  **(95% CI)** | **p-value** |
| **BMI categories** |  |  |  |  |  |  |  |  |  |  |  |  |  |  |  |  |
| Below 18.5 | 43  (1.2%) | 23.3% | 1 | - | 79  (1.1%) | 35.4% | 1 | - | 219  (1.1%) | 50.7% | 1 | - | 341 (1.1%) | 43.7% | 1 | - |
| 18.5–24.9 | 1,356  (37.4%) | 28.4% | 1.20  (0.73–1.96) | 0.466 | 2,568  (37.3%) | 41.5% | 1.11  (0.84–1.47) | 0.466 | 7,059  (36.1%) | 47.9% | 0.96  (0.85–1.08) | 0.500 | 10,983  (36.5%) | 44.0% | 1.00  (0.90–1.12) | 0.933 |
| 25.0–29.9 | 1,111  (30.6%) | 29.3% | 1.27  (0.77–2.10) | 0.340 | 2,414  (35.1%) | 45.0% | 1.19  (0.90–1.58) | 0.232 | 6,915  (35.4%) | 52.2% | 1.05  (0.90–1.18) | 0.442 | 10,440  (34.7%) | 48.1% | 1.09  (0.98–1.22) | 0.129 |
| 30.0 and above | 611  (16.9%) | 29.8% | 1.23  (0.74–2.04) | 0.423 | 1,316  (19.1%) | 47.3% | 1.23  (0.93–1.64) | 0.154 | 4,064  (20.8%) | 56.4% | 1.10  (0.98–1.24) | 0.118 | 5,991  (19.9%) | 51.7% | 1.13  (1.02–1.27) | **0.026** |
| Unknown | 505  (13.9%) | 24.2% | 1.25  (0.72–2.18) | 0.434 | 503  (7.3%) | 36.2% | 1.31  (0.95–1.79) | 0.097 | 1,291  (6.6%) | 50.7% | 1.12  (0.98–1.29) | 0.104 | 2,299  (7.7%) | 41.7% | 1.15  (1.02–1.27) | **0.025** |
| **Test provider** |  |  |  |  |  |  |  |  |  |  |  |  |  |  |  |  |
| Test provider 1 | 143 (3.9%) | 22.4% | 1 | - | 241 (3.5%) | 29.9% | 1 | - | 520  (2.7%) | 51.7% | 1 | - | 904  (3.0%) | 41.3% | 1 | - |
| Test provider 2 | 1,517 (41.8%) | 28.3% | 0.94  (0.64–1.39) | 0.769 | 2,745 (39.9%) | 44.8% | 1.16 (0.90–1.51) | 0.257 | 8,100  (41.4%) | 54.3% | 0.97  (0.86–1.09) | 0.546 | 12,362  (41.1%) | 49.0% | 1.02  (0.91–1.13) | 0.765 |
| Test provider 3 | 623 (17.2%) | 29.9% | 1.10 (0.77–1.58) | 0.602 | 1,212 (17.6%) | 43.0% | 1.13 (0.88–1.45) | 0.349 | 3,495  (17.9%) | 48.5% | 0.85  (0.76–0.95) | **0.004** | 5,330  (17.7%) | 45.0% | 0.92  (0.83–1.02) | 0.111 |
| Test provider 4 | 1,182 (32.6%) | 29.1% | 1.08 (0.73–1.59) | 0.701 | 2,426 (35.3%) | 42.9% | 1.29  (1.00–1.68) | 0.054 | 6,703  (34.3%) | 49.3% | 0.96  (0.85–1.08) | 0.463 | 10,311  (34.3%) | 45.5% | 1.04  (0.94–1.16) | 0.453 |
| Test provider 5 | 161 (4.4%) | 20.5% | 0.69 (0.43–1.10) | 0.121 | 256 (3.7%) | 46.5% | 1.16 (0.88–1.53) | 0.295 | 730  (3.7%) | 52.5% | 0.91  (0.80–1.03) | 0.130 | 1,147  (3.8%) | 46.6% | 0.95 (0.84–1.06) | 0.346 |

Particip.^1^ = Number of participants; Seropositive^2^= Percentage of seropositive participants; PRR = Prevalence rate ratio; COVID symptoms = Symptoms compatible with COVID-19; CI = confidence interval; P < 0.05 was considered significant (in bold).
