# Supplementary material for: Nationwide increases in anti-SARS-CoV-2 IgG antibodies between October 2020 and March 2021 in the unvaccinated Czech population
Source: Commun Med (Lond). 2022 Mar 1;2:19. doi: 10.1038/s43856-022-00080-0 (PMC9053194; doi:10.1038/s43856-022-00080-0)
Supplement: Supplementary file 3 — Supplementary Data 2 [file 43856_2022_80_MOESM3_ESM.docx]

**Supplementary Table 1.** Source data underlying Figure 1: Dynamics of the COVID-19 pandemic in the Czech Republic and seroprevalence in the first phase of the PROSECO study between October 2020 and March 2021

| **Period** (year-week) | **% of persons with SARS-CoV-2 antibodies in the PROSECO study** | **Cumulative % of persons positively tested for SARS-CoV-2 (PCR or antigen) among persons aged 18+** | **Cumulative % of persons positively tested for SARS-CoV-2 (PCR only) among persons aged 18+** | **% of positive PCR tests among all PCR tests provided (all ages)** | **Number of deaths from all causes according to the Czech Statistical Office (all ages)** |
| --- | --- | --- | --- | --- | --- |
| 2020-09 | - | 0.0% | 0.0% | 27.3% | 2,360 |
| 2020-10 | - | 0.0% | 0.0% | 4.2% | 2,294 |
| 2020-11 | - | 0.0% | 0.0% | 6.4% | 2,295 |
| 2020-12 | - | 0.0% | 0.0% | 7.0% | 2,325 |
| 2020-13 | - | 0.0% | 0.0% | 6.4% | 2,297 |
| 2020-14 | - | 0.0% | 0.0% | 5.2% | 2,320 |
| 2020-15 | - | 0.1% | 0.1% | 4.8% | 2,244 |
| 2020-16 | - | 0.1% | 0.1% | 2.7% | 2,125 |
| 2020-17 | - | 0.1% | 0.1% | 1.6% | 2,036 |
| 2020-18 | - | 0.1% | 0.1% | 0.8% | 2,109 |
| 2020-19 | - | 0.1% | 0.1% | 0.8% | 1,999 |
| 2020-20 | - | 0.1% | 0.1% | 0.8% | 2,026 |
| 2020-21 | - | 0.1% | 0.1% | 1.1% | 1,919 |
| 2020-22 | - | 0.1% | 0.1% | 0.9% | 1,939 |
| 2020-23 | - | 0.1% | 0.1% | 1.2% | 2,135 |
| 2020-24 | - | 0.1% | 0.1% | 1.8% | 1,983 |
| 2020-25 | - | 0.1% | 0.1% | 2.4% | 2,022 |
| 2020-26 | - | 0.1% | 0.1% | 4.2% | 2,082 |
| 2020-27 | - | 0.1% | 0.1% | 3.5% | 2,111 |
| 2020-28 | - | 0.1% | 0.1% | 2.7% | 1,999 |
| 2020-29 | - | 0.1% | 0.1% | 2.7% | 1,989 |
| 2020-30 | - | 0.2% | 0.2% | 3.7% | 2,088 |
| 2020-31 | - | 0.2% | 0.2% | 3.2% | 2,227 |
| 2020-32 | - | 0.2% | 0.2% | 3.3% | 2,166 |
| 2020-33 | - | 0.2% | 0.2% | 3.6% | 2,187 |
| 2020-34 | - | 0.2% | 0.2% | 4.0% | 2,104 |
| 2020-35 | - | 0.3% | 0.3% | 4.4% | 2,033 |
| 2020-36 | - | 0.3% | 0.3% | 5.4% | 2,038 |
| 2020-37 | - | 0.4% | 0.4% | 8.1% | 2,089 |
| 2020-38 | - | 0.5% | 0.5% | 10.1% | 2,220 |
| 2020-39 | - | 0.6% | 0.6% | 11.2% | 2,392 |
| 2020-40 | 0.0% | 0.8% | 0.8% | 14.6% | 2,381 |
| 2020-41 | 9.0% | 1.2% | 1.2% | 23.5% | 2,609 |
| 2020-42 | 9.1% | 1.8% | 1.8% | 27.2% | 2,946 |
| 2020-43 | 18.6% | 2.6% | 2.6% | 31.2% | 3,647 |
| 2020-44 | 18.6% | 3.5% | 3.5% | 31.0% | 4,226 |
| 2020-45 | 31.2% | 4.3% | 4.3% | 29.8% | 4,238 |
| 2020-46 | 26.7% | 4.7% | 4.7% | 23.7% | 3,852 |
| 2020-47 | 42.4% | 5.1% | 5.0% | 22.0% | 3,424 |
| 2020-48 | 34.5% | 5.3% | 5.3% | 19.8% | 3,169 |
| 2020-49 | 42.8% | 5.6% | 5.6% | 19.9% | 3,100 |
| 2020-50 | 38.6% | 6.0% | 5.9% | 21.6% | 3,104 |
| 2020-51 | 44.9% | 6.4% | 6.3% | 23.0% | 3,242 |
| 2020-52 | 42.9% | 6.9% | 6.7% | 28.7% | 3,214 |
| 2020-53 | 39.2% | 7.6% | 7.4% | 38.7% | 3,518 |
| 2021-01 | 43.8% | 8.5% | 8.2% | 34.3% | 3,812 |
| 2021-02 | 45.4% | 9.1% | 8.7% | 26.4% | 3,722 |
| 2021-03 | 45.7% | 9.6% | 9.1% | 25.5% | 3,565 |
| 2021-04 | 42.2% | 10.0% | 9.5% | 25.2% | 3,402 |
| 2021-05 | 46.6% | 10.5% | 9.9% | 25.7% | 3,247 |
| 2021-06 | 48.4% | 11.1% | 10.4% | 27.3% | 3,404 |
| 2021-07 | 47.7% | 11.7% | 10.9% | 29.2% | 3,452 |
| 2021-08 | 49.3% | 12.5% | 11.6% | 31.3% | 3,599 |
| 2021-09 | 50.3% | 13.3% | 12.3% | 31.5% | 3,898 |
| 2021-10 | 50.7% | 14.1% | 13.0% | 32.1% | 3,987 |
| 2021-11 | 51.3% | 14.7% | 13.5% | 30.4% | 3,796 |
| 2021-12 | 53.7% | 15.2% | 14.0% | 27.0% | 3,427 |
| 2021-13 | 58.1% | 15.6% | 14.3% | 24.3% | 3,369 |
| 2021-14 | - | 15.8% | 14.5% | 20.0% | 2,889 |
| 2021-15 | - | 16.1% | 14.7% | 16.4% | 2,577 |
| 2021-16 | - | 16.2% | 14.9% | 14.0% | - |
| 2021-17 | - | 16.4% | 15.0% | 11.4% | - |
| 2021-18 | - | 16.5% | 15.1% | 9.3% | - |
| 2021-19 | - | 16.5% | 15.2% | 6.4% | - |
| 2021-20 | - | 16.6% | 15.2% | 3.1% | - |
| 2021-21 | **-** | 16.6% | 15.2% | 2.3% | **-** |
